# Preoperative chemotherapy reduces the accumulation of indocyanine green around colorectal liver metastases for use in fluorescence-guided surgery

**DOI:** 10.1007/s00464-025-12034-3

**Published:** 2025-09-11

**Authors:** Okker D. Bijlstra, Tom H. Dijkhuis, Friso B. Achterberg, Alexander Broersen, Jouke Dijkstra, Mats I. Warmerdam, Rutger B. Henrar, Jacobus Burggraaf, A. Stijn L. P. Crobach, Rutger-Jan Swijnenburg, Peter J. K. Kuppen, Alexander L. Vahrmeijer, J. Sven D. Mieog

**Affiliations:** 1 Department of Surgery, Leiden University Medical Center, Leiden, The Netherlands; 2 Department of Surgery, UMC, Location Vrije Universiteit, Amsterdam, the Netherlands; 3 Department of Radiology, Leiden University Medical Center, Leiden, The Netherlands; 4 Centre of Human Drug Research, Leiden, The Netherlands; 5 Department of Pathology, Leiden University Medical Center, Leiden, The Netherlands

**Keywords:** Fluorescence-guided surgery, Colorectal liver metastases, Indocyanine green, Near-infrared fluorescence imaging

## Abstract

**Background:**

Near-infrared fluorescence imaging using intravenously administered indocyanine green (ICG) improves colorectal liver metastases (CRLM) surgery by enhancing lesion detection and real-time tumor margin assessment. However, ICG accumulates in hepatocytes around CRLM with high variance between patients. This study evaluates the effects of tumor and patient characteristics on ICG accumulation using a standardized imaging and analysis workflow.

**Methods:**

This single-center study included patients with CRLM who received 10 mg of ICG intravenously 24 h before surgery. Resected lesions were sliced in 5–10-mm-thick sections and immediately imaged for standardized fluorescence analysis. Fluorescence parameters were compared based on chemotherapy treatment, tumor response, tumor size and superficiality, and degree of steatosis. Associations between the patient and tumor characteristics and fluorescence parameters were determined while correcting for confounders.

**Results:**

Thirty-two lesions from 32 patients were analyzed. Lesions from chemotherapy-pretreated patients exhibited a lower mean signal fluorescence intensity (MSFI, 0.23 vs. 0.65 a.u.; *p* = 0.002) and signal-to-background ratio (SBR, 2.28 vs. 6.08; *p* < 0.001) than lesions from patients without pretreatment. Tumor size correlated positively with MSFI (*p* = 0.003), SBR (*p* = 0.02), and maximum intensity (*p* < 0.001). After correcting for the other characteristics, chemotherapy showed statistically significant association with the fluorescence parameters. The tumor superficiality, degree of steatosis, and response to chemotherapy had no statistically significant associations with the fluorescence parameters.

**Conclusion:**

Neoadjuvant chemotherapy significantly lowers ICG accumulation around CRLM resulting in suboptimal contrast. To optimize fluorescence-guided surgery protocols for chemotherapy-pretreated patients, future research should focus on adjusting ICG dose and timing and exploring specific fluorescence tumor-targeting imaging agents.

Near-infrared (NIR) fluorescence imaging using indocyanine green (ICG) augments colorectal liver metastases (CRLM) surgery [1–7] through (1) improved detection of preoperatively identified lesions, (2) the intraoperative identification of otherwise occult lesions, and (3) enabling intraoperative tumor margin assessment [2, 6–9]. Therefore, NIR ICG fluorescence-guided imaging has been implemented as standard of care during CRLM surgery in several hospitals. ICG is typically administered intravenously with varying doses of 0.3–0.5 mg/kg or a standard dose of 5—25 mg. Also, the timing of the ICG injection varies widely from 1 to 14 days before surgery [2, 4, 6, 10–12]. ICG is actively transferred from the plasma into hepatic parenchymal cells, and is excreted solely by the biliary tract without being metabolized [13, 14]. It clears from healthy tissue but remains in the tumor periphery, enabling the identification of lesions up to approximately 8-mm subsurface with NIR imaging [1, 9, 15]. During and after resection, both the resection specimens and the wound bed can be inspected for residual fluorescent signal which indicates narrow or positive tumor margins [6].

Tumor margin evaluation is of great importance as tumor-positive resection margins have a negative effect on the 5-year survival rate, recurrence-free survival, and disease-free survival of patients undergoing CRLM surgery [16–21]. Recently, we showed in a prospective multicenter trial the benefit of ICG fluorescence to achieve high radical resection rates (92.4%) in minimally invasive CRLM surgery even in centers new to the technique [7].

Despite the benefits for the use of ICG in CRLM surgery, the relatively high false-positive identification (10–20%) and false-positive resection rates (~ 10–16%) cause reluctance for wide implementation of the technique [7, 22]. Furthermore, the accumulation of ICG around CRLM and in the normal liver parenchyma varies per patient and lesion leading to high variation in fluorescence intensities hampering the interpretation of the fluorescent signal during surgery. Several patient and tumor characteristics may influence these differences (for instance, neoadjuvant chemotherapy, hepatic comorbidities, and location (i.e., superficial versus deeper located lesions) and size of the lesion). However, the influence of these characteristics on the accumulation of ICG surrounding CRLM has not yet been investigated to the best of our knowledge.

In this study, we quantitatively analyzed the effect of patient and tumor characteristics on ICG fluorescence around CRLM utilizing a recently published standardized analysis method [23].

## Methods

### Patients

This study was approved by the Dutch Medical Ethical Committee Leiden, Delft, Den Haag, with study number B19.056. Patients undergoing open or minimally invasive surgery for colorectal liver metastases between June 2019 and December 2022 who received 10 mg of ICG approximately 24 h before surgery as per the hospital’s standard of care protocols were included in this single-center study. Patients were excluded when there was insufficient ‘healthy’ liver tissue (100 mm^2^) in the resected specimen to perform the analysis. All patients provided informed consent.

### Imaging procedure

**Fig. 1:**
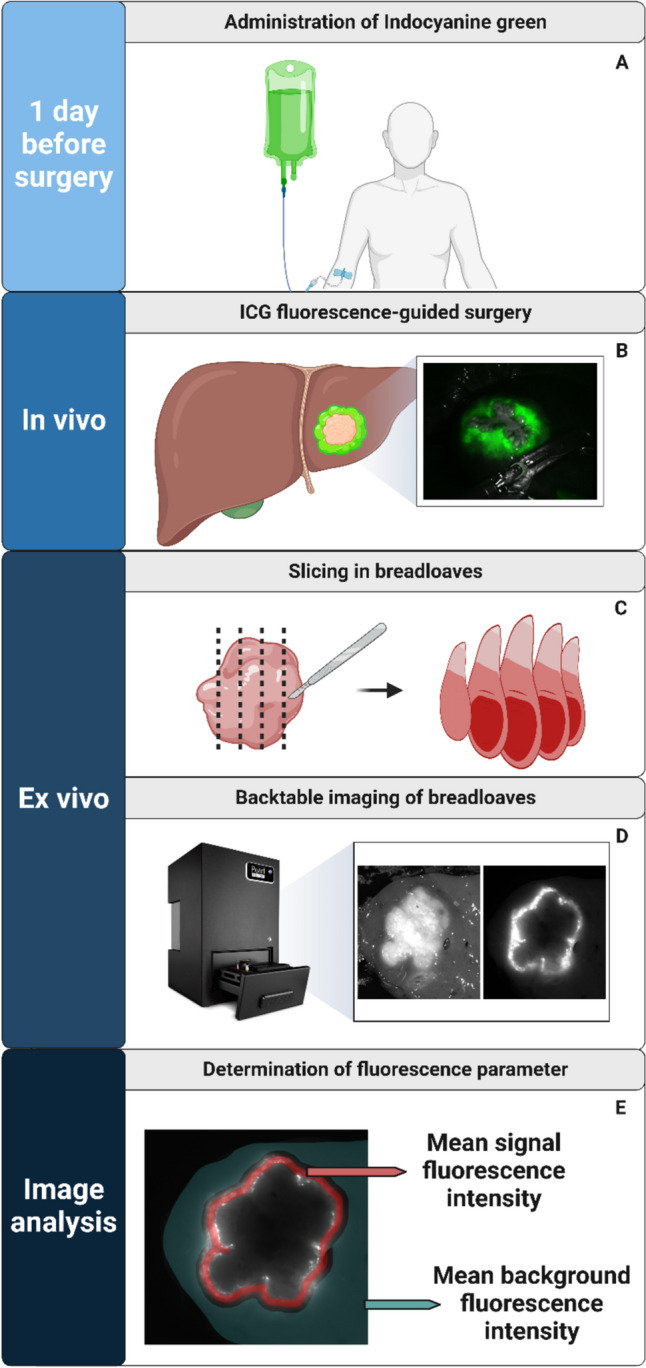
Schematic overview of the methodology. One day before surgery patients received 10-mg indocyanine green intravenously (**A**). During surgery ICG was used as guidance (**B**). After surgery, the resected specimen was sliced in bread loaves (**C**). These bread loaves are then images with a back-table imaging system (**D**), with semi-automatic determination of the fluorescence parameters (**E**). *Created with BioRender.com*

During surgery, ICG was utilized as a qualitative marker for CRLM according to the hospital’s standard of care protocol (Fig. 1B). Immediately post-surgery, a standardized ex vivo imaging workflow was implemented [23]. At the pathology department, the pathologist inked the resection plane to preserve the orientation of the tumor in relation to the resection plane. The samples were then sectioned into 5–10-mm-thick bread loafs. (Fig. 1C).

Each bread loaf was imaged using the PEARL Trilogy imaging system (LI-COR Biotechnology, Lincoln, Nebraska, USA) (Fig. 1D). The exposure time was set automatically. Two images were then acquired: a white light image and an image with a peak excitation wavelength of 785 nm and a peak emission wavelength of 820 nm, resulting in a NIR fluorescence image. The images were captured at a resolution of 85 µm and dimensions of 1300 × 964 pixels.

### Image analysis

For the image analysis, the protocol described previously was followed to objectify the analysis [23]. After imaging of the specimen, the tumor and bread loaves were annotated in the white light images in QuPath (version 0.5.1) [24]. Quantitative analysis of the images was performed in MeVisLab (version 3.4.1, MeVis Medical Solutions AG and Fraunhofer MEVIS). To analyze the fluorescent rim, the contrast between the fluorescence in the rim was compared to the background fluorescence. As described in our proposed protocol, the rim fluorescence, i.e., signal fluorescence, was defined as the fluorescence up to 3 mm from the tumor. The background was defined as the fluorescence in the remaining liver tissue from 5 mm from the tumor (Fig. 1E). The bread loaf located at the center of the tumor was selected for image analysis to reduce the risk on selection bias.

### Data analysis and statistics

#### Patient characteristics and fluorescence parameters

Patient characteristics (e.g., age, sex, steatosis hepatis) and fluorescence parameters were used to compare the different tumors and patients in this study. Pretreatment of patients was noted if the last cycle of chemotherapy started within three months before surgery. Besides chemotherapy (e.g., FOLFOX and CapOx), targeted therapy (i.e., Bevacizumab) was also noted as this therapy completes the neoadjuvant systemic treatment regimen. Steatosis hepatis was scored retrospectively by a dedicated pathologist (SC). The Hematoxylin–Eosin-stained slides were scored between no steatosis, mild steatosis (5–33%), moderate steatosis (34–66%), and severe steatosis (> 66%) according to Brunt et al. [25]. The fluorescence parameters included the mean signal fluorescence intensity (MSFI), the mean background fluorescence intensity (MBFI) and the signal-to-background ratio (SBR). The values were computed by calculating the mean fluorescence intensity in the aforementioned predefined areas. Moreover, the size of the area from which the MBFI was calculated was computed. In addition, the maximum intensity value of the fluorescent image was determined and exported. All fluorescence parameters were calculated automatically by the software. After generation, the parameters were manually checked. Outlying background fluorescence intensities were recalculated when stasis (unspecific accumulation of ICG) of ICG was present in the bread loaf by selecting a representative area for the background fluorescence. Moreover, the background fluorescence intensity was recalculated when there was less than 100 mm^2^ liver tissue by manually adding background fluorescent areas in other bread loaves from the same specimen.

#### In-depth fluorescence data analysis

First, an analysis was performed to investigate the relation of the grade of steatosis hepatis on the accumulation of ICG in the liver. Then, the effect of treatment with neoadjuvant chemotherapy and the superficiality of the tumor on the fluorescence parameters was determined. For the analysis of the effect of neoadjuvant chemotherapy, patients who did and who did not receive chemotherapy within 3 months prior to surgery were divided into two groups. For a more in-depth explorative analysis to investigate the potential predictability of the effect of chemotherapy on the accumulation of ICG around CRLM, the radiological response of CRLM on the chemotherapy was stratified. The response was scored in line with the RECIST 1.1 criteria [26]. Tumors were scored separately. Stable disease was defined as an increase in tumor size of maximum 20% or decrease of maximum 30%, partial response as at least 30% decrease in tumor size, complete response when the lesion disappeared, and progressive disease when the tumor increased at least 20% in size. No stratification based on targeted therapy was performed as pretreatment regimens not always include targeted agents. For the analysis of the superficiality of the tumors, lesions were divided into capsular or subcapsular tumors. The effect of the tumor size was investigated by performing a correlation analysis between the different fluorescence parameters and the tumor size. The tumor size was defined as the maximal radiological diameter. At last, the association per tumor and patient characteristic with the fluorescence parameters was determined by correcting for the effect of the other tumor and patient characteristics on the fluorescence parameters.

#### Statistics

Microsoft Excel (Microsoft Windows) and SPSS Statistics software version 25.0 (IBM) were used for data analysis. Means with standard deviations were used for the normal distributed parameters and medians with the interquartile range (IQR) were used for the non-normal distributed data. A one-way ANOVA test was performed on the means of normal distributed parameters for the comparison of the groups. For non-normal distributed parameters, the Mann–Whitney *U* statistical test was performed for this comparison. The Pearson Chi-Square test was performed to test the statistical significance from the categorical variables. Furthermore, the relation between steatosis hepatis and the fluorescence background intensity was tested by performing a Kruskal–Wallis analysis based on the steatosis groups. Spearman’s correlation coefficients were computed to test the correlation between the tumor size and the separate fluorescence parameters. Finally, multiple linear regression analyses were performed to test the associations of the different patient and tumor-specific parameters with the fluorescence data. The four fluorescence parameters (MSFI, MBFI, SBR, and maximum fluorescence intensity) were all used as dependent variables while pretreatment, superficiality of the tumor, and size of the tumor were used as tested variables. No statistical test was performed for the explorative analysis of the radiological response of chemotherapy due to the small group sizes. Graphical data were generated with GraphPad Prism Version 9.01 (San Diego, California USA). Statistical outcomes were considered significant when the p value was lower than 0.05.

## Results

### Patients

NIR fluorescence images were acquired from 42 patients between June 2019 and February 2022. After exclusion of patients with insufficient peritumoral healthy tissue for the quantitative analysis or with a primary tumor other than colorectal, 32 patients were included for the final analyses. The descriptive statistics of these patients is presented in Table 1. Ten patients received neoadjuvant chemotherapy, while the remaining underwent surgery without upfront chemotherapy. Eight of patients who received chemotherapy also received targeted therapy. Seven patients received CapOx, varying from 2 to 7 cycles and three patients received 6–8 cycles of FOLFOX with bevacizumab. In total, 32 resected tumors were analyzed. Twenty-two tumors (22/32) were in the capsule, and ten lesions were located subcapsular. Four livers showed moderate steatosis, 19 livers showed mild steatosis and nine livers showed no steatosis in the liver. The mean diameter of the tumors was 29.9 ± 15.4 mm.

**Table 1:** Descriptive statistics and fluorescence parameters of the complete cohort, the cohort based on pretreatment, and the cohort based on tumor superficiality

	All patients (*N* = 32)	Neoadjuvant chemotherapy (*N* = 10)	No neoadjuvant chemotherapy (*N* = 22)	*p*-value	Capsular tumor (*N* = 22)	Subcapsular tumor (*N* = 10)	*p*-value
Sex ratio (M:F)	19:13	3:7	16:6	0.022*	16:6	3:7	0.022*
Age at surgery (years)	63.2 ± 8.9	61.60 ± 10.68	63.86 ± 8.21	0.516	62.95 ± 10.08	63.60 ± 6.13	0.853
BMI	25.3 ± 3.0	25.26 ± 3.52	25.38 ± 2.81	0.922	25.76 ± 2.97	24.41 ± 2.96	0.244
Neoadjuvant chemotherapy							
Yes	10^†^	NA	NA	NA	7	4	0.488
No	22	NA	NA		15	6	
Superficiality metastasis							
Capsular	22	6	16	0.488	NA	NA	NA
Subcapsular	10	4	6		NA	NA	
Location of primary tumor							
Cecum	2	1	1	0.146	1	1	0.351
Ascending colon	3	2	1		2	1	
Transverse colon	1	1	0		0	1	
Descending colon	6	1	5		3	3	
Sigmoid	12	3	9		11	1	
Rectum	8	2	6		5	3	
Steatosis hepatis							
No steatosis	9	1	8	0.121	7	2	0.795
Mild (5–33%)	19	7	12		11	8	
Moderate (33–66%)	4	2	2		4	0	
Severe (> 66%)	0	0	0		0	0	
Tumor diameter (cm)	2.99 ± 1.54	2.27 ± 1.77	3.32 ± 1.34	0.073	2.97 ± 1.48	3.05 ± 1.76	0.892
Signal fluorescence intensity (a.u.)	0.45 [0.23, 0.87]	0.23 [0.13, 0.32]	0.65 [0.40, 0.99]	0.002*	0.60 [0.28, 0.89]	0.31 [0.17, 0.77]	0.309
Background fluorescence intensity (a.u.)	0.104 [0.060, 0.135]	0.114 [0.059, 0.144]	0.104 [0.062, 0.128]	0.714	0.115 [0.062, 0.137]	0.072 [0.057, 0.112]	0.180
Signal-to-Background ratio	4.62 [2.49, 8.43]	2.28 [1.75, 2.86]	6.08 [4.16, 10.37]	< 0.001*	4.62 [2.42, 9.45]	4.31 [3.01, 5.93]	0.903
Maximum fluorescence intensity (a.u.)	2.34 ± 0.89	1.64 ± 0.60	2.66 ± 0.82	0.001*	2.50 ± 0.87	2.00 ± 0.90	0.148

*a.u*. arbitrary units, *BMI* body mass index

*Statistically significant difference, ^†^Eight patients received targeted therapy

Values are *n*, mean ± standard deviation, or median [Interquartile range]

#### Fluorescence parameters

The median background fluorescence intensity of all lesions was 0.104 [0.060, 0.135] arbitrary units (a.u.) and the median signal fluorescence intensity 0.45 [0.23, 0.87] a.u., resulting in a median SBR of 4.62 [2.55, 8.10]. The mean maximum fluorescence intensity was 2.34 ± 0.89 a.u.

#### Effect of steatosis hepatis on background fluorescence in the liver

**Fig. 2:**
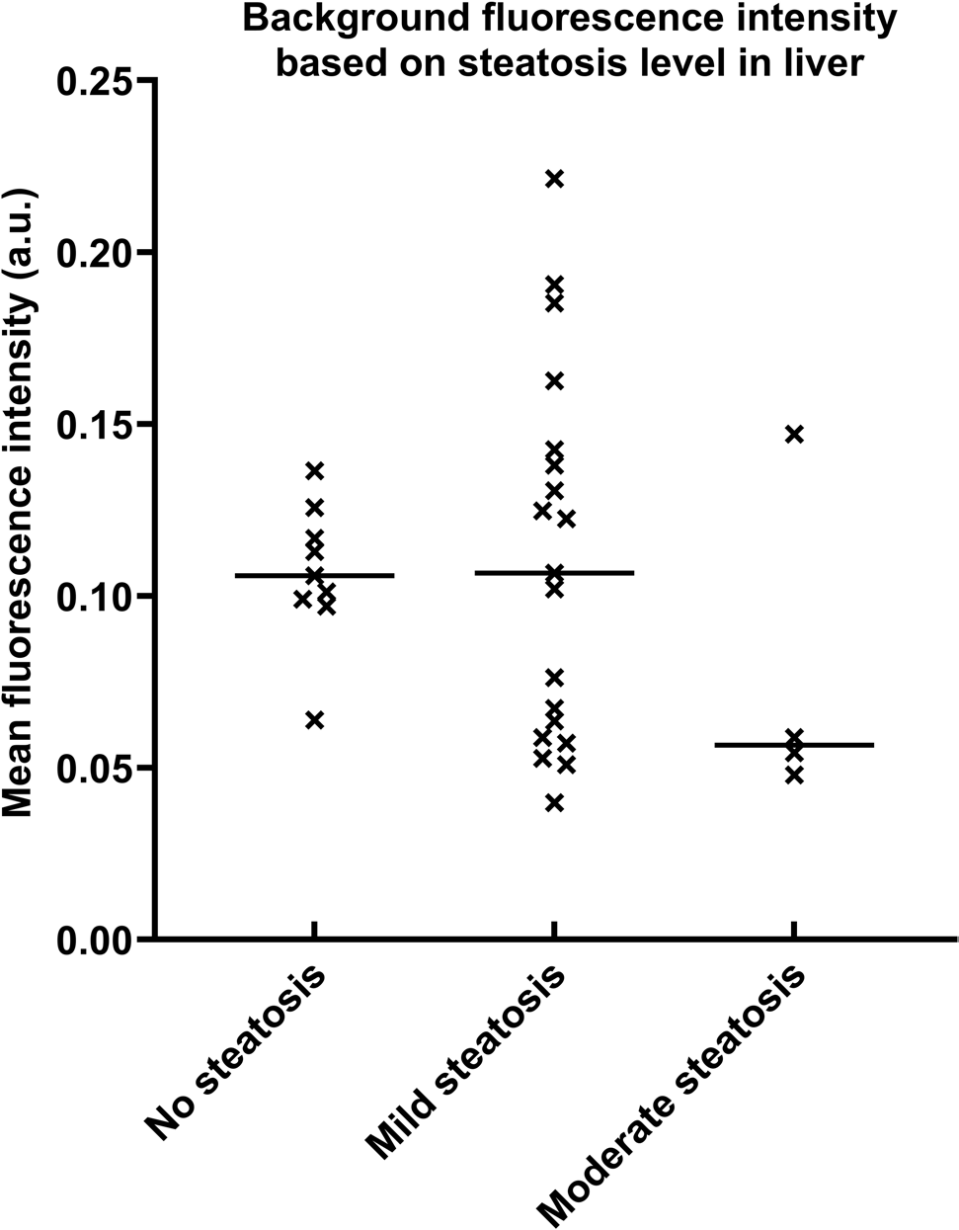
Visualization of the background fluorescence intensity stratified by the steatosis grade in the liver. This figure shows the individual background fluorescence intensities in the liver and the median per steatosis group. The moderate steatosis group showed a lower median fluorescence intensity in the liver without reaching statistical significance. *a.u.* arbitrary units

The moderate steatosis group (*N* = 4) showed the lowest median MBFI (0.057 a.u.). Mild steatosis (0.106 a.u; *N* = 19) and no steatosis (0.107 a.u.; *N* = 9) showed similar background fluorescence values. No statistically significant difference in MBFI between the three groups was found (*p* = 0.39). The results of this analysis are shown in Fig. 2.

#### Neoadjuvant chemotherapy affects ICG accumulation around the CRLM

**Fig. 3:**
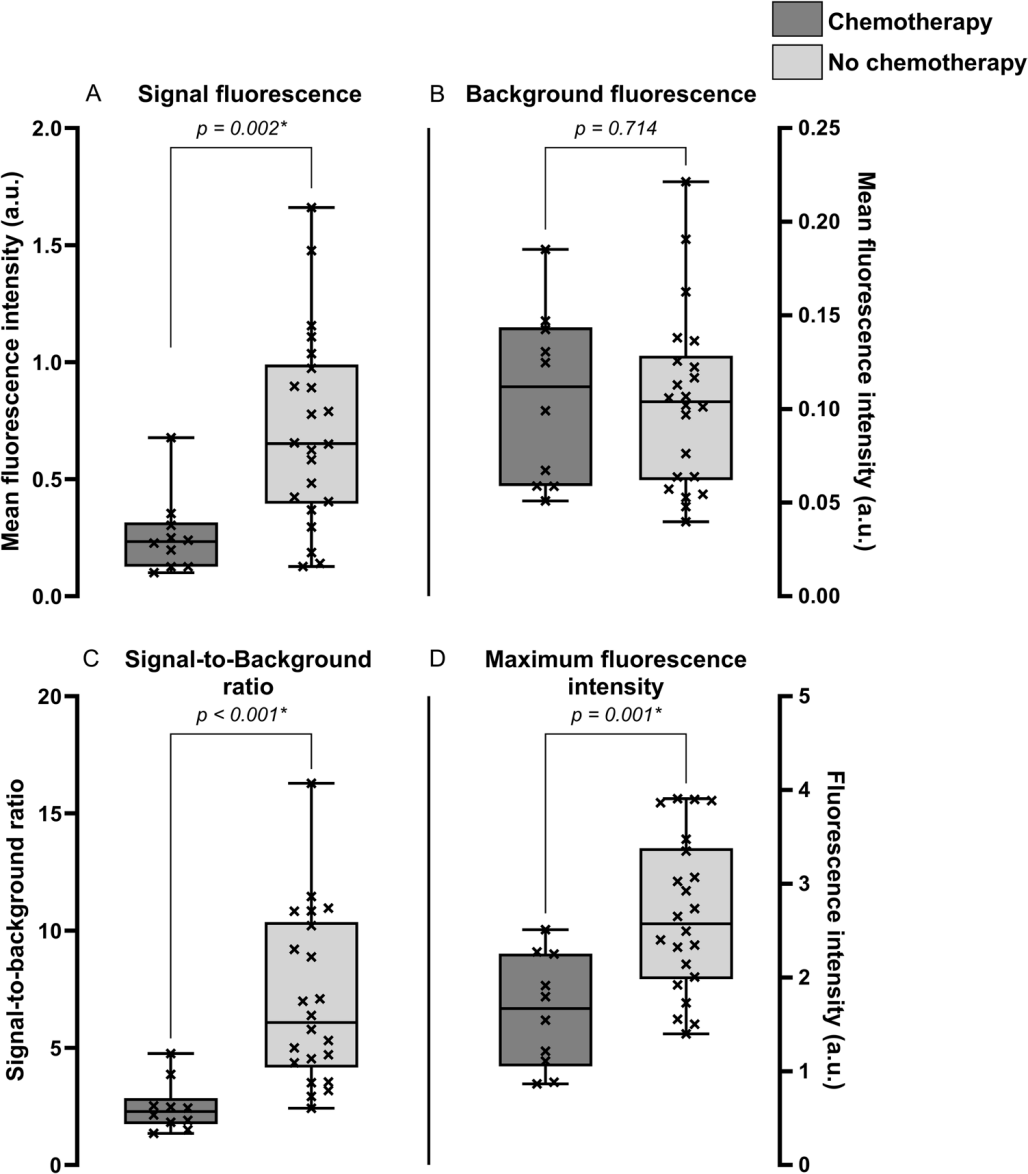
Visualization of the fluorescence parameters stratified by neoadjuvant chemotherapy. This figure shows in **A**–**D** the median, interquartile ranges, minima, maxima, and all separate data points for the patient groups with and without pretreatment. Statistically significant lower signal fluorescence, background fluorescence, and signal-to-background ratios are found for patients who received neoadjuvant chemotherapy. * *p* value is statistically significant, *a.u.* arbitrary units

**Fig. 4:**
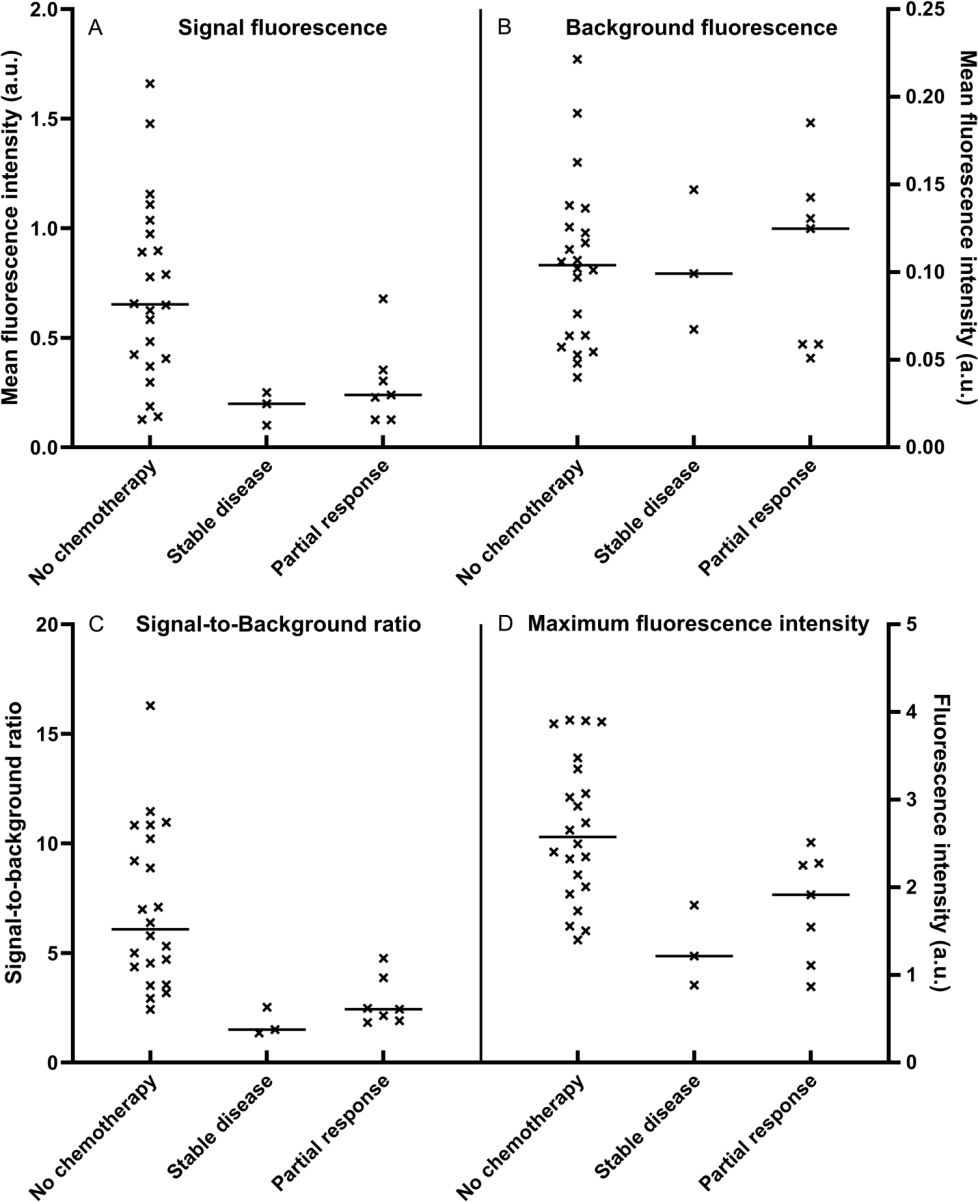
Fluorescence parameters stratified by radiological response. This figure shows in **A**–**D** the median and all separate data points stratified by the radiological response after chemotherapy treatment. Patient with tumors which were categorized as having stable disease showed lower MSFI, SBR, and maximum fluorescence intensity then patients who were categorized as partial response or who did not receive chemotherapy. *a.u.* arbitrary units, *MSFI* mean signal fluorescence intensity, *SBR* signal-to-background ratio

The groups with and without neoadjuvant chemotherapy were comparable for age at surgery, tumor superficiality, steatosis in the liver, tumor diameter, and MBFI (Table 1). Maximum fluorescence intensity (1.64 ± 0.60 a.u. vs 2.66 ± 0.82 a.u.; p = 0.001), MSFI (0.23 [0.13, 0.32] a.u. vs 0.65 [0.40, 0.99] a.u.; *p* = 0.002), and SBR (2.28 [1.75, 2.86] vs 6.08 [4.16, 10.37]; *p* < 0.001) were significantly higher in the group of patients that did not receive neoadjuvant chemotherapy (Fig. 3A–D). From the 10 patients treated with neoadjuvant therapy, seven patients had tumors with a radiological partial response and three patients had stable disease. The fluorescence parameters were stratified for an explorative analysis based on the radiological response. A decrease in MSFI (0.65 a.u. vs 0.24 and 0.20 a.u.), SBR (6.08 vs 2.43 and 1.50), and maximum fluorescence intensity (2.57 a.u. vs 1.91 and 1.21 a.u.) was observed for the groups with patients that received chemotherapy (Fig. 4).

#### Effect of tumor superficiality on fluorescence parameters

**Fig. 5:**
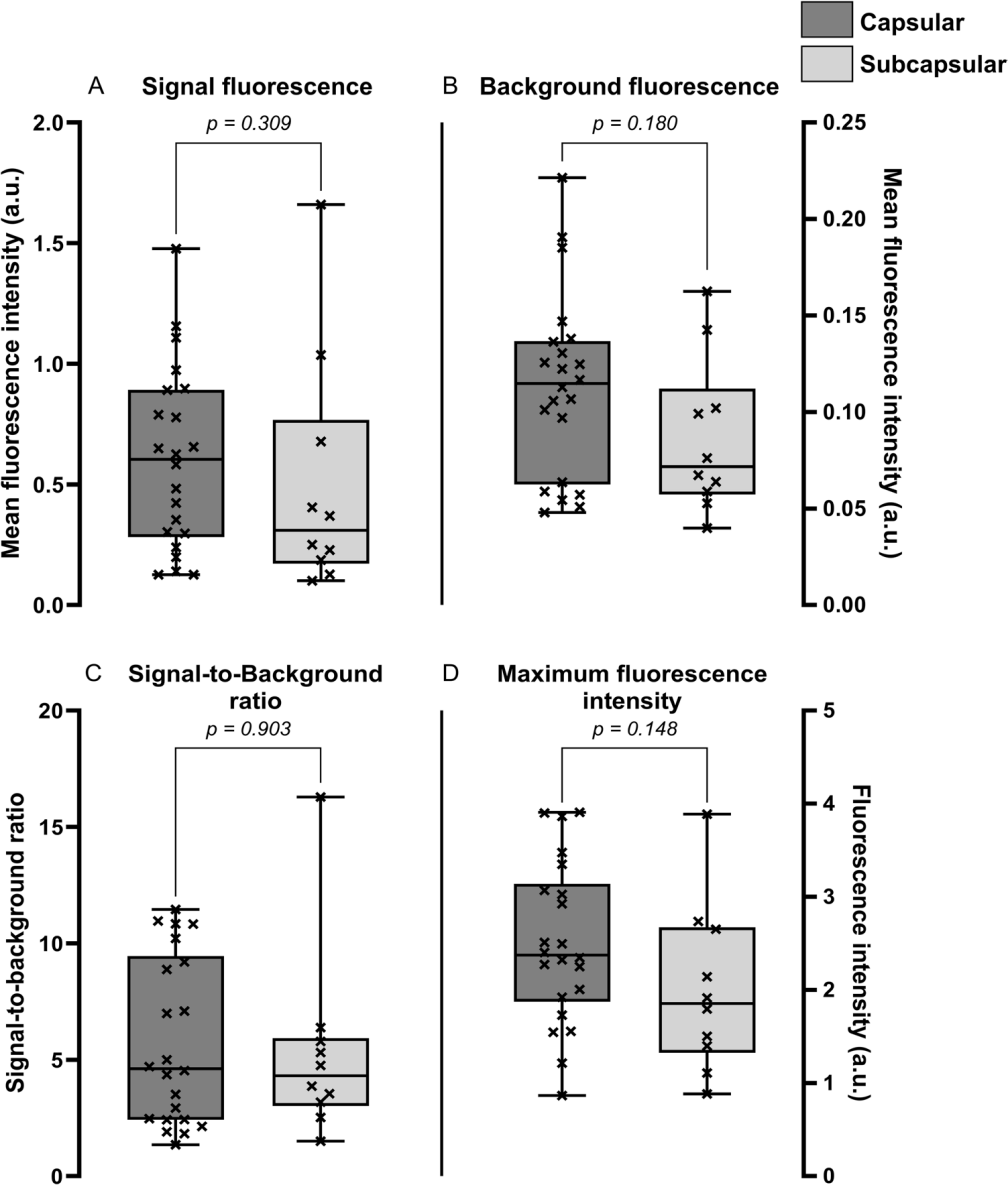
Visualization of the fluorescence parameters stratified by tumor superficiality. This figure shows in **A**–**D** the median, interquartile ranges, minima, maxima, and all separate data points for the patient groups with capsular and subcapsular tumors. No statistically significant differences were found between both groups. *p value is statistically significant, *a.u.* arbitrary units

The subcapsular group had a significantly higher female:male ratio (*p* = 0.02) than the capsular group. No other differences in patient characteristics were observed between both groups. The fluorescence parameters showed no statistical differences between both groups (Fig. 5A–D). The results of this comparison are summarized in Table 1.

#### Effect of tumor size on fluorescence parameters

**Fig. 6:**
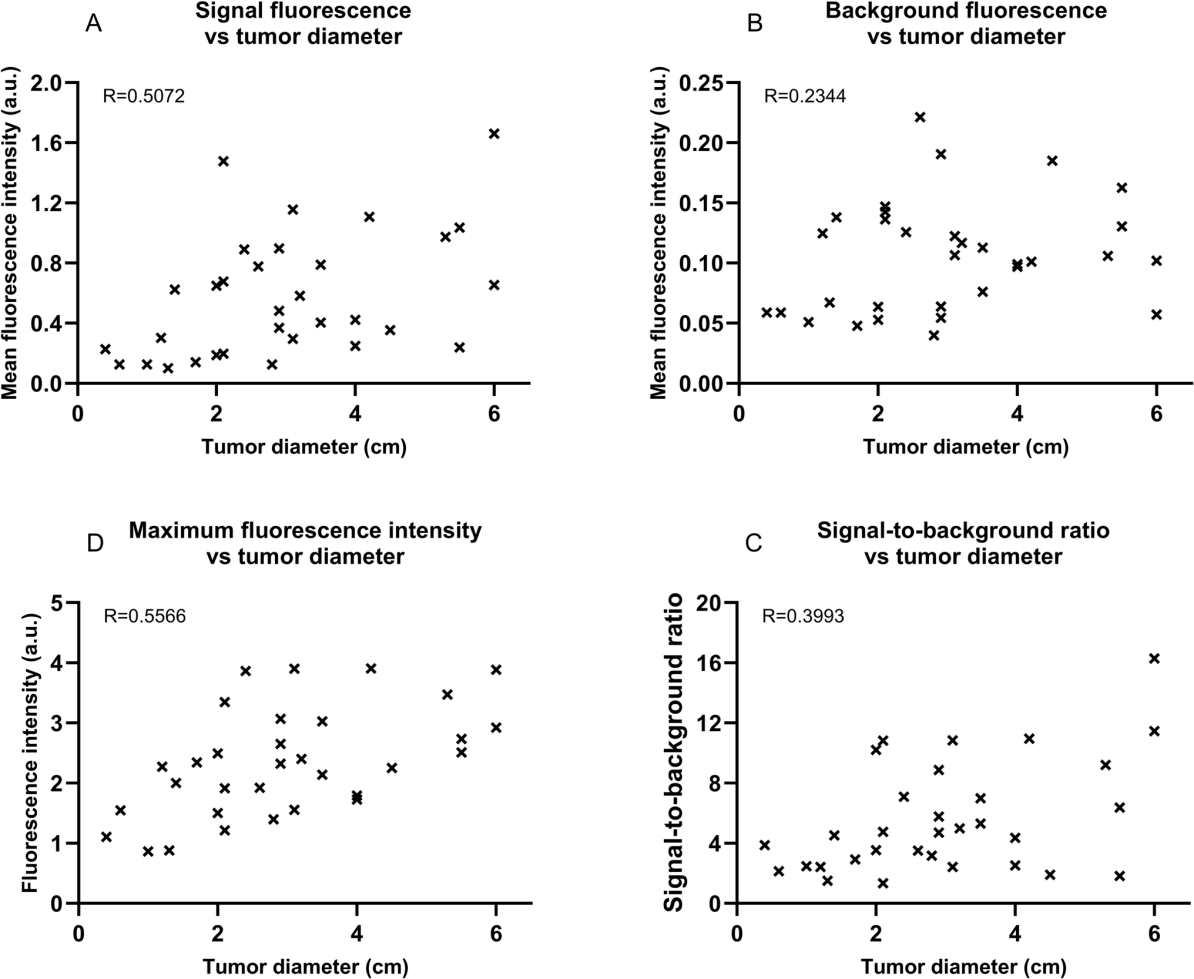
Visualization of the fluorescence parameters correlated with tumor size. This figure shows in **A**–**D** the correlation between tumor size and the fluorescence parameters. The tumor size was statistically significantly correlated with signal fluorescence, signal-to-background ratio, and maximum fluorescence intensity. All three showed a positive correlation with the tumor size (increase in fluorescence parameters with increasing tumor size). *a.u.* arbitrary units

The analysis of the correlations between the tumor size and the separate fluorescence parameters showed a statistically significant positive correlation between tumor size and MSFI (*R* = 0.5072, *p* = 0.003), SBR (*R* = 0.3993, *p* = 0.02), and the maximum fluorescence intensity (*R* = 0.5566, *p* < 0.001). This indicates that increasing tumor size correlates with a higher MSFI, SBR, and maximum fluorescence intensity. No significant correlation was observed for the background fluorescence which resulted in a p value of 0.20. Scatterplots to visualize the correlations between the tumor size and the MSFI, MBFI, SBR, and the maximum fluorescence intensity are illustrated in Fig. 6A–D.

#### Multiple linear regression analysis: association of patient and tumor characteristics with fluorescence parameters

The MSFI, SBR, and maximum fluorescence intensity, showed, after correcting for the influence of the other characteristics on the fluorescence parameters, a statistically significant association with pretreatment with chemotherapy of *p* = 0.02, *p* = 0.004, and *p* = 0.01, respectively. These associations were related to a decrease of 0.36 in MSFI, 3.8 in SBR, and 0.72 in maximum fluorescence intensity. The size of the tumor showed no statistically significant association with the MSFI (*p* = 0.06) and SBR (*p* = 0.05). However, a statistically significant association was observed between tumor size and the maximum fluorescence intensity (*p* = 0.006). This association was related to a 0.25 increase in maximum fluorescence intensity for a 1 cm increase in tumor size. Both the tumor size (*p* = 0.14) and pretreatment with chemotherapy (*p* = 0.43) did not show statistically significant associations with MBFI. The superficiality of the tumor showed no association with any of the four fluorescence parameters.

## Discussion

This study was performed to investigate the effect of chemotherapy treatment, tumor superficiality and size, and steatosis hepatis on the peritumoral fluorescent rim of ICG around CRLM and the background fluorescence signal of ICG in the liver. The use of neoadjuvant chemotherapy was associated with a significant decrease of the accumulation of ICG close to CRLM resulting in lower contrast between the signal and background fluorescence. The similar MBFIs in surrounding non-cancerous liver tissue suggest that the clearance of ICG by non-cancerous liver parenchyma was not affected by previous chemotherapy. The explorative comparison between the radiological response and the fluorescence parameters suggests that the radiological response does not influence the accumulation of ICG around the tumors in these patients. However, no final conclusions can be drawn based on the current small sample size. Larger tumors showed a correlation with a higher signal fluorescence, maximum fluorescence intensity and SBR. However, after correcting for confounders (pretreatment with chemotherapy and superficiality of the metastasis), no statistically significant association between the size of the tumor and the signal fluorescence (*p* = 0.06) and SBR (*p* = 0.05) was observed. This suggests that pretreatment with chemotherapy potentially affected the results of the earlier correlation analysis. Increased hepatis steatosis did not correlate with a higher background fluorescence intensity. The highest level of steatosis hepatis (moderate steatosis) showed a lower background fluorescence intensity (0.057 a.u.) compared to mild and no steatosis (0.107 and 0.106 a.u.). This result should however be interpreted with care due to the small sample size.

The accumulation of ICG around CRLM may be attributed to the presence of immature hepatocytes around CRLM that exhibit impaired biliary clearance, because of lower expression of multidrug resistance-associated proteins 2 (MDRP2) [3]. A future study should also analyze differences in MDRP2 expression between patients. However, other mechanisms may also play a role in the accumulation of ICG [27]. One of these mechanisms is halting tumor growth or achieving a reduction in the size of a growing tumor which could mitigate the compression exerted on the adjacent liver parenchyma, potentially enhancing the liver’s ability to clear ICG. The finding of reduced contrast between the fluorescent rim and the background fluorescence in non-cancerous liver tissue corresponds to earlier observations in previous studies [4, 28]. In contrast to the findings of the current study, one study identified a higher background fluorescence intensity in the liver as the reason for the reduced contrast in hepatocellular carcinoma patients with reduced liver function as a result of preoperative chemotherapy or liver cirrhosis [4]. Moreover, decreased enhancement of CRLM by ICG in patients pretreated with chemotherapy is also reported [28]. However, it was not reported if this was due to increased background fluorescence or decreased signal fluorescence intensity. Although the slight increase in background fluorescence intensity in the liver (0.104 a.u. to 0.114 a.u.) after chemotherapy, the decreased accumulation of ICG around CRLM appears to be the main reason for the reduced contrast ratio. The tumor size did not show statistically significant associations with higher MSFI and SBR after correcting for the effect of preoperative chemotherapy. However, it is recommended that the tumor size is considered in future follow-up studies as a potential effector on fluorescence parameters to further investigate its effect. The standardized approach of the image analysis has been recently published in a study which showed improved inter- and intraobserver variabilities compared to a standard manual analysis method, highlighting the workflow’s robustness for fluorescence image analysis [23]. Therefore, utilizing this approach strengthens the conclusions of the current study compared to the other studies. Therefore, we believe the decreased SBRs are predominantly caused by a decreased accumulation of ICG around CRLM.

The findings of this study indicate that patients pretreated with chemotherapy could potentially benefit from a different dose of ICG and a different interval of ICG administration to surgery compared to non-pretreated patients. Specifically, to improve standard operating procedures with the use of fluorescence guidance, i.e., scanning the liver surface for identification of metastases and potential detection of novel lesions and the assessment of the resection margins. Current international guidelines regarding ideal dose and timing of ICG in CRLM surgery are consensus-based and lack substantial scientific evidence [29]. Moreover, a recent Delphi study clearly depicted the significance of evidence-based guidelines for ideal dose and timing of ICG administration [30]. Therefore, our group initiated a new multicenter study (MIMIC-II, NL-OMON56540) to investigate the influence of varying time intervals and dose adjustment of ICG administration prior to surgery on the in vivo and ex vivo fluorescence parameters in patients who are pretreated with chemotherapy.

An alternate approach to overcome the problem of low SBRs in neoadjuvant chemotherapy treated patients and to reduce false-positive tumor detection and resection rates is the use of (additional) tumor-specific imaging agents. SGM-101 is a tumor-specific fluorescent dye targeting CEA for CRLM, which is excited at ~ 700 nm [31]. This dye showed promising results in patients with both primary colorectal cancer and CRLM and is currently being evaluated in an international multicenter phase III trial (SGM-CLIN03, NCT03659448) in primary colorectal carcinoma [32–34]. ICG and SGM-101 are excited on distinct wavelengths and both target a different area during CRLM surgery, hence the combination of both compounds could aid in the interpretability of the resection margins. To investigate the feasibility of the combination of these two fluorescent dyes for CRLM surgery our group initiated a separate clinical feasibility study (FOCUS GREEN, NCT05965817).

Although this study showed promising results, several limitations should be addressed. First, due to the small sample size and single-center nature of the study conclusions should be drawn with caution. Only 10 patients received preoperative chemotherapy, with varying treatment regimens. Due to the small sample size a subgroup analysis in chemotherapy patients was not possible. Further analysis after expansion of the dataset should be performed for more detailed research on the effect of the various tumors (e.g., tumor biology) and patient characteristics in structured and powered subgroup analyses. Moreover, the correlation of intraoperative and postoperative imaging data should be analyzed, as well as the correlation of imaging with clinical outcomes and resection margin status. The variability of the fluorescence parameters was relatively high between patients decreasing the predictability of the accumulation of ICG around CRLM. In an expanded cohort, the predictability of the behavior of ICG around CRLM based on the radiological response of lesions on chemotherapy should be further explored. Moreover, in-depth histopathological analysis could be performed to further investigate the effect of the tumor biology on ICG accumulation on microscopic level. At last, the effect of the addition of targeted therapy as well as the effect of the different types of therapy regimens (e.g., FOLFOX-B, FOLFIRI, CapOx) could be further investigated.

## Conclusion

In this study, we found that patients treated with neoadjuvant chemotherapy had significant lower signal-to-background ratios and mean fluorescent intensities than patients that did not receive neoadjuvant therapy. Future research should optimize fluorescence-guided surgery protocols for chemotherapy-pretreated patients, including ICG dose and timing adjustments and exploring tumor-specific imaging agents.

## Data Availability

Data and code developed in this study are available upon reasonable request to the corresponding author.
